# Ergonomic evaluation of the Senhance® robotic system in minimally invasive gynecologic procedures versus conventional laparoscopy: an exploratory study focusing on surgeon’s muscle activity

**DOI:** 10.1007/s00404-025-08292-0

**Published:** 2026-01-16

**Authors:** Bernhard Krämer, Jürgen Andress, Leonhard Wohlmeiner, Robert Seibt, Benjamin Steinhilber

**Affiliations:** 1 Department of Women’s Health, University Hospital Tübingen, Calwerstraße 7/6, 72076 Tübingen, Germany; 2 Institute of Occupational and Social Medicine and Health Services Research, University Hospital Tübingen, University of Tübingen, Wilhelmstraße 27, 72074 Tübingen, Germany

**Keywords:** Robotic-assisted surgery, Ergonomics, Surface electromyography, Static muscle demands, Musculoskeletal discomfort, Work-related musculoskeletal disorders

## Abstract

**Purpose:**

Primary: To evaluate whether robotic-assisted laparoscopic surgery using the Senhance® Surgical System has the potential to reduce muscular demands compared to conventional laparoscopy. Secondary: To verify that the novel eye-tracking feature for zoom and the selection of functions in the Senhance® System menu is not associated with increased eyestrain or neck strain.

**Methods:**

In a within-subject design, 2 experienced surgeons performed 11 robot-assisted and 12 conventional laparoscopic procedures. Muscular demands were monitored throughout surgical procedures by assessing the muscle activity via bipolar surface electromyography of seven muscles of the lower back, shoulder–neck, and forearms. Surgeons’ head, arm, and torso posture was assessed by gravimetrical position sensors. Furthermore, musculoskeletal discomfort, mental and physical workload, task difficulty and working precision were rated by the surgeons. In addition, a ten-item eyestrain questionnaire was administered after each surgical procedure.

**Results:**

Four out of seven muscles were relieved when working with Senhance®. Only in the left shoulder–neck area there was a statistically significant increase in muscle activation associated with robotic-assisted surgery. Changes in surgeons’ posture related to the surgical technique corresponded to the changes in muscle activation. Furthermore, surgeons reported no musculoskeletal discomfort under both conditions and similar levels of workload (mental and physical), and task difficulty. Working precision was subjectively rated to be better during standard laparoscopy. No eyestrain occurred during any of the procedures.

**Conclusion:**

This exploratory study identified the potential of the Senhance® Surgical System for ergonomic improvements and indicated no impairments by the novel eye-tracking feature on muscle demands and eyestrain. Follow-up studies with larger and more diverse indications are needed that also consider clinical outcomes, which were not part of the present study.

**Supplementary Information:**

The online version contains supplementary material available at 10.1007/s00404-025-08292-0.

## Introduction

Work-related musculoskeletal disorders (WRMSD) among surgeons performing minimally invasive surgery are common [1, 2]. The most often affected body regions are the neck, back, shoulders, wrists, hands, and thumbs [3–5]. The exposure to static and awkward positioning, repetitive motions, hyperflexion of the cervical spine, mainly related to the workplace layout and equipment design [6] together with high workloads are considered as physical risk factors, significantly contributing to the development of WRMSD [2]. A survey published in 2020 among the members of the Israeli Society of Endoscopic Surgery further indicates a dose–response relationship between years of exposure and WRMSD. The authors reported a WRMSD prevalence of 12% before beginning the practice of laparoscopic surgery compared to 78% after 15–20 years of practice [7].

In many countries, the need for medical care will increase due to demographic trends towards an aging population and at the same time, many surgeons will retire in the coming years. Consecutively, a shortage of surgically active medical doctors [8–10] as well as associated restrictions in patient care will represent future risk for global health systems.

In this regard, measures for preventing or reducing work-related demands on surgeons performing minimally invasive surgery are urgently required. Robotic-assisted minimally invasive surgery has been shown to provide ergonomic benefits for the surgeon compared to standard minimally invasive procedures [11, 12] without negatively affecting patient outcomes [13, 14]. However, commercially available robotic platforms differ in their design (instrument handles, console layout) and the way they are operated, so that it is not possible to generalize about positive ergonomic effects. Ideally, all robotic systems should be subjected to an ergonomic assessment as this might help to identify the systems that fit the approach of the particular surgical team and discipline and to justify the investment.

In this study, we aimed to evaluate the ergonomic potentials of the Senhance® Surgical System (Asensus Surgical Inc., Durham, NC, USA). The primary outcome of this exploratory study was muscular strain in the lower back, lower arm, and shoulder neck area, while performing robotic-assisted laparoscopic surgeries (RALS) vs. conventional laparoscopic surgery (CLS). As secondary outcomes, body postures, cardiovascular demands (heart rate), perceived physical discomfort, and workload (physical and mental) were assessed. Since this surgical robot includes a novel feature allowing the surgeons to control the endoscope by eye/head movements, eye and neck strain were additionally evaluated as secondary outcome.

## Materials and methods

### Subjects and sample size

Two surgeons who are experts in minimally invasive gynecology participated in the present study. Inclusion criteria were having already performed ≥ 10 RALS with the Senhance® Surgical System to achieve a stable competence level in the learning curve, the (physical and cognitive) ability to work the full shift, voluntary participation, and a signed written informed consent. The study was approved by the Ethics Committee of the Medical Faculty of the University of Tübingen, Germany (262/2018BO1) and has been registered on ClinicalTrials.gov (Identifier: NCT06109753).

No sample size calculation has been performed for this exploratory study. Due to the completely different body postures between the two surgical techniques (sitting vs. standing), a total case number of ten RALS and ten CLS were considered sufficient for detecting a difference in muscular demands related to the surgical technique.

### Procedures

**Fig. 1:**
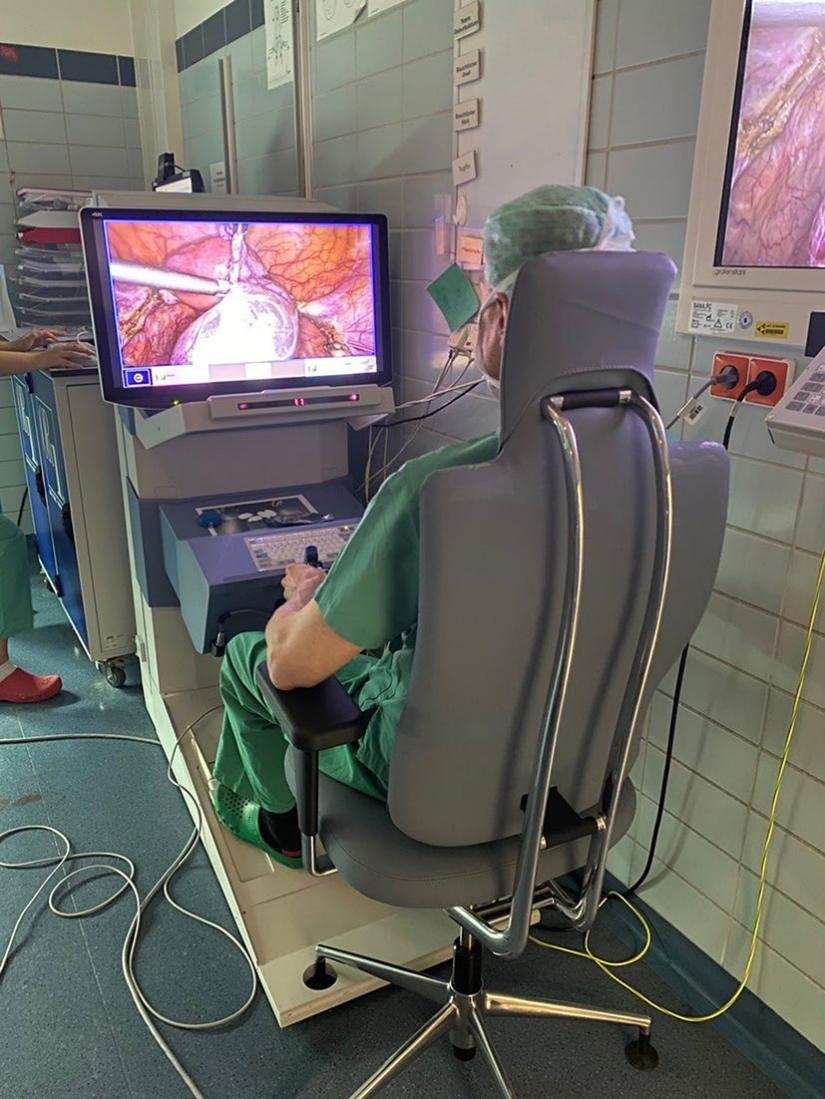
Set up of the Senhance® Surgical System

**Fig. 2:**
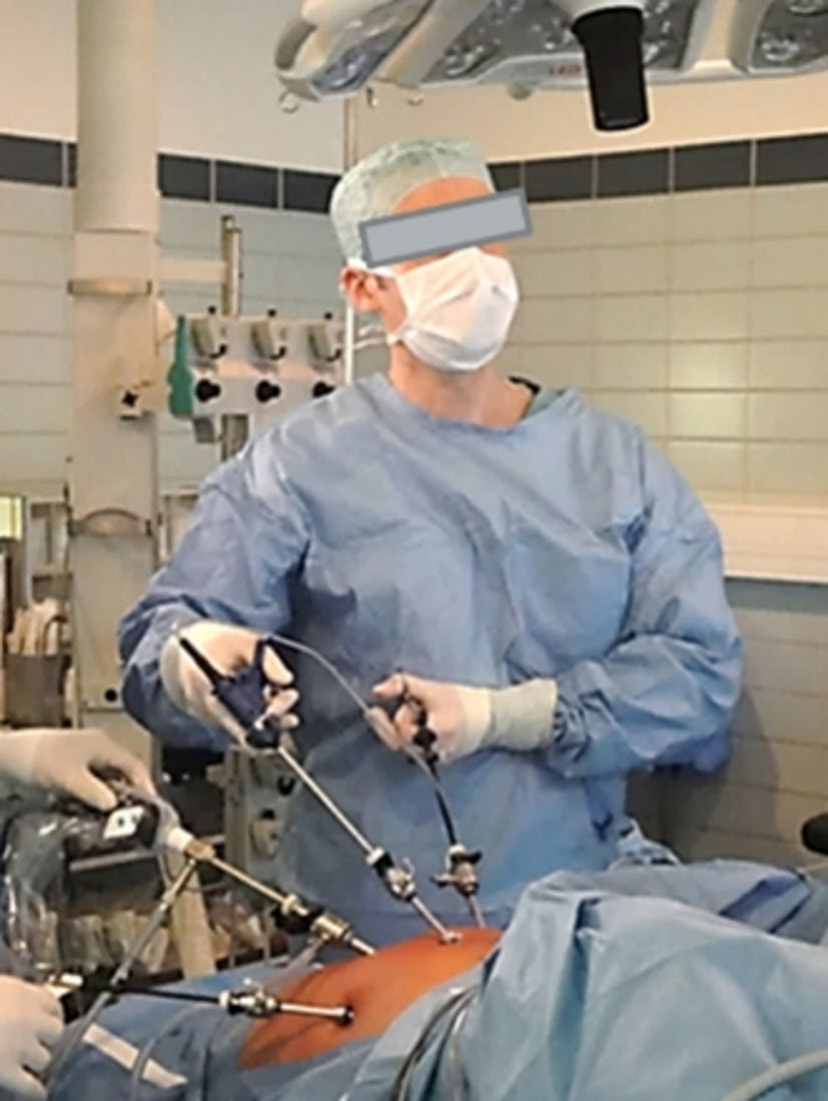
Set up of the conventional laparoscopic procedures

According to their daily clinical routine, each of the two surgeons performed at least five RALS and five CLS from June 2023 to November 2023. Therefore, the order of surgeries and measurement days took place as per schedule of the department and was not randomized. The RALS were conducted with the Senhance® Surgical System (Asensus Surgical Inc., Durham, NC, USA) (Fig. 1) which will be explained in detail in the next section. CLS were performed as usual according to the routine setting of the department (Fig. 2).

After connecting the surgeons to the measurement equipment, including surface electromyography (sEMG), electrocardiography (ECG), and posture, reference contractions for sEMG normalization were recorded. Data were collected continuously using a PS12-II device (THUMEDI® GmbH & Co. KG, Thum, Germany) during the real surgical procedures. The PS12 device weighs 800 g and was worn in a bag on the surgeon’s body. This approach has already been successfully used previously without interfering with the surgical procedures [15, 16]. The perceived level of musculoskeletal discomfort of the surgeon was queried orally every 20 min and at the very end of the surgical procedure with respect to affected body areas. Immediately after completing the surgical procedure, the surgeons were asked to rate their perceived work load, the difficulty of the surgery, perceived working precision, and eyestrain. After each RALS, the surgeons were additionally asked to assess usability of the robotic system.

### Robotic surgical system

The Senhance® Surgical System is a modular robotic platform with an open console unlike the well-known da Vinci® robotic surgical system (Intuitive Surgical, Inc., Sunnyvale, CA, USA). Surgeons are sitting in front of an open 2D/3D HD monitor (78 cm screen diagonal) that is integrated into the console on a rotatable and height adjustable chair with back, head, and lower arm support. To get full 3D vision, the surgeons have to wear 3D glasses. The position of the chair is fixed to the console by means of docking points on the floor to optimize 3D visualization and enable eye tracking by keeping the surgeon’s eyes around 85 cm from the monitor.

The interface between the robotic device and the surgeon is through hand pieces that exactly mimic the handle of a standard laparoscopic instrument and provides haptic feedback with regards to the force required to cut and touch tissue. A special new feature of Senhance is the “eye-tracking camera control” that is achieved by an eye tracker located under the monitor. The eye-tracking control allows eye movement-controlled pan and zoom features for the camera. Before 3D vision and eye tracking can be activated, the surgeon has to calibrate these functions by following a short algorithm that is displayed on the screen ensuring that the position of the eyes as well as the height of the monitor and the chair are aligned. From an economic point of view, Senhance® is potentially less expensive as it is equipped with reusable robotic instruments that are designed to endure multiple sterilization cycles compared to current DaVinci systems where the instruments can only be used for a certain number of procedures according to a pre-defined lifetime [17, 18].

### Measurements and data analysis

#### Primary outcome

##### Muscular strain

Bipolar surface electromyography (sEMG) was used to measure muscle activities of the left erector spinae muscle (ES), bilateral descending trapezius muscles (TD), right extensor digitorum muscle (ED) and right flexors carpi radialis muscles (FCR) and bilateral splenius capitis muscle (SC). This selection aimed to include muscles from body areas that are prone to musculoskeletal complaints in minimally invasive procedures (TD and SC for the shoulder–neck region, ED and FCR for the hand–arm region and ES for the lower back region). In addition, this selection accounts for the dominance of the right arm and hand under CLS conditions: the left ES stabilizes the torso in situations where the dominant right arm maintains an arm abduction or anteversion posture; the most workload regarding instrument use occurs in the dominant right hand represented by ED and FCR analysis. Finally, the SC was measured bilaterally as this muscle is an important head extensor and is involved in head lateral flexion and rotation. The demands of this muscle may change when using Senhance® with the zoom function which is controlled by eye, head, or torso movements (leaning forward or backwards to zoom in and out). The skin over these muscles was cleaned with abrasive paste (Nuprep skin preparation gel, Weaver and Company, USA) and shaved if there was excessive body hair. Electrodes were self-sticking silver/silver chloride (Ag/AgCl) electrodes with an active diameter of 15 mm and an inter-electrode distance (center to center) of 25 mm. The EMG signal was differentially amplified, transmitted, filtered (high-pass filter, second order, 4 Hz; low-pass filter, 11th order, -3 dB@1300 Hz), sampled (4096 Hz), analyzed, and stored (PS12-II, THUMEDI® GmbH & Co. KG, Thum, Germany; physical resolution 24bit; common-mode rejection ration > 98 dB, effective sum of noise < 0.5 µV RMS; linearity ± 0.1 dB at 30-1200 Hz). The data were real-time transformed in the frequency domain (1024-point fast Fourier transformation, 250-ms Bartlett window, 50% overlap) and digitally filtered (high-pass filter, 11th order, 16 Hz). Interfering powerline noise was removed by an average filter (11th order, 50 Hz and its first seven harmonics, 4-Hz bandwidth was replaced by spectral neighbors). The root mean square of electrical activity (RMS [μV]) was real-time calculated from the power spectrum (250-ms moving window, 50% overlap) and stored synchronously with the raw data. The RMS of each muscle was normalized to the RMS obtained during a muscle specific isometric reference contraction. Typically, sEMG is normalized to the muscle activity obtained during an isometric maximum voluntary contraction (MVC) and expressed as percent of the muscle’s maximum voluntary electrical activation (%MVE). This procedure is a standard in studies using sEMG and can be generally considered as safe in terms of increased risk for muscle injury. However, as MVC puts the highest strain on a muscle for safety reasons (surgeon and patient), we decided not to apply MVC reference contraction. Instead, we used submaximal reference contractions for sEMG normalization to decrease the variance. Details about the procedures of reference contractions can be found in the supplemental material (SUPPLEMENT A). The normalized RMS values were further used to calculate the three outcome measures representing muscular demands: the 50th (median activity), 10th (static activity), and 90th (peak activity) percentiles electrical activity of the surgical procedures. All of these three outcomes are associated with the risk of WRMSDs [19, 20].

#### Secondary outcomes

##### Neck, arm, and torso posture

2D gravimetric position sensors (sampling rate 8 Hz; resolution of 0.1° and 125 ms in time; maximum static error of 0.5° against the perpendicular; maximum repetition error of 0.2°) continuously recorded inclination angles with respect to the absolute perpendicular (gravitational axis) as flexion and lateral flexion. The sensors were attached to the skin at the spinous process of thoracic vertebrae one (T1), the middle of the lateral part of both upper arms and the forehead using double-sided adhesive tape (25 mm × 5 m; 3 M transparent Medical Standard, Top Secret®, Gesellschaft für Haarästhetik mbH, Fürth, Germany). The sensors measured the inclination in degree towards the perpendicular line with respect to the sagittal plane (x-value) and frontal plane (y-value). Because the sensors are attached directly to the body and there are no flat surfaces due to musculature, this inevitably results in values that do not correspond to the actual position of the individual body segment. This means, for example, that an upper arm hanging vertically downwards will still show an inclination because the sensor cannot be attached directly to the bone. Therefore, each measurement signal during the surgical procedures was offset-adjusted according to a reference posture recorded prior to the experimental conditions by subtraction. During the reference posture, the inclination angles of sensors were recorded while each subject was standing in their common upright standing posture with the arms hanging down vertically and looking straight ahead for about 10 s. The median of the most stable 3–5 s within this period was defined as the reference. These posture data were then processed to estimate joint angles from sensor-pair difference. In the Supplementary material (SUPPLEMENT B), there is a detailed description of the sensor-pair-differences used to determine the joint angles in the frontal and sagittal plane of the head and shoulders. Trunk flexion and lateral flexion, however, were estimated only by the sensor placed on T1. Data were given as median (50th percentile), 10th, and 90th percentiles of each surgery, representing the middle and peak postures, respectively. After each measured surgery, the reference posture was applied again to rule out possible sensor displacement during the surgical procedure.

##### Perceived musculoskeletal discomfort

Surgeons rated their level of perceived musculoskeletal discomfort using an 11-point Likert scale (0 = no discomfort, 10 = maximum discomfort). The scale has been validated for pain assessment, showing excellent reliability (intraclass coefficient of 0.95) and good to excellent validity compared with a visual analog scale or verbal rating scale (Pearson correlation coefficient of 0.94 or 0.93) [21]. Although it has not been specifically validated for the assessment of musculoskeletal complaints, it is commonly used in occupational settings [22]. Subjects were also asked to indicate the location of the discomfort (neck, shoulder, upper arm, elbow, forearm, wrist, finger(s), upper back, lower back, or other). These ratings were used to calculate the relative frequency of perceived discomfort as the number of discomfort ratings divided by the number of discomfort ratings per surgical procedure and reported as a percentage. In addition, the mean intensity of discomfort was calculated in relation to the affected body part.

##### Perceived physical and mental workload

For assessing perceived physical and mental* w*orkload, the physical and mental demand dimensions of the NASA-TLX questionnaire were used. Each dimension is rated on a 21-graded scale [0–20] from very low to very high. This questionnaire is an established and well-accepted tool in ergonomic research [23].

##### Heart rate

As an indicator of cardiovascular demands, the electrical activity of the heart was recorded using electrocardiography (ECG) by two pre-gelled Ag/AgCl electrodes placed ~ 5 cm cranial and ~ 3 cm left-lateral from the distal end of the sternum and over the anterior to mid-axillary line at the fifth left rib. ECG signals were continuously recorded (sample rate 1000 Hz) and processed in real time to calculate the heart rate (HR [bpm]) and was given as median per surgical procedure.

##### Eyestrain

For the assessment of perceived eyestrain related to the surgeries, a standardized questionnaire with ten items was used [24]. Each item contained a symptom such as “dry eyes” and the surgeon was requested to rate how much this symptom was present during the surgery using a seven-point Likert scale (0 = none, 6 = most severe).

#### Data to further characterize the surgical performance

Several supplemental outcomes were assessed to adequately describe working under CLS and RALS conditions. The two surgeons subjectively rated the difficulty of each surgery and their perceived working precision regarding the applied surgical technique by a visual analog scale [0–100 mm]. Finally, the total surgical times of each surgical procedure without incision, suturing, and preparing the technical devices were recorded.

### Statistical analysis

Outcome variables were visually inspected for extreme values, plausibility, and missing data. Normal distribution was verified according to proposed limits in standard errors of skewness and kurtosis [25]. Means and standard deviations or boxplots including median and the upper and lower quartiles or frequencies were used to describe the results. Data were analyzed using JMP 16.2.0 (SAS Inc. Cary, NC, USA) with an alpha level of 0.05.

The primary outcomes (50th, 10th, and 90th percentile of the electrical muscle activity) and the secondary outcomes (posture and heart rate) were analyzed using a repeated analysis of variance (R-ANOVA) with subjects as random effect and the surgical technique as within-effect. This statistical analysis has been shown to be robust against violation of the normal distribution [26].

The outcomes perceived physical discomfort, mental and physical workload, task difficulty and precision, eyestrain as well as surgical time were analyzed in a descriptive manner.

## Results

### Participants and surgeries

Finally, 23 surgeries were included with 6 RALS and 7 CLS performed by surgeon one and 5 RALS and 5 CLS by surgeon two. Due to the small sample size and to maintain anonymity, the characteristics of the surgeons are given in ranges (Table 1). The exact sequence of operations and measurement days for each surgeon were documented and are provided as supplemental material as well as the performed surgical procedures (SUPPLEMENT C).

**Table 1:** Characteristics of study population

Gender	2 males
Age [15-year interval]	40–55
Body weight [15-kg interval]	70–85
Body height [15-cm interval]	180–195
Regular physical activity [N]	Yes = 2 No = 0
Minimum number of conventional laparoscopies before study participation	> 300
Minimum number of procedures with the Senhance® Surgical System before study participation	≥ 10

#### Primary outcome: muscle activity

In Table 2, an overview of the three characteristics of muscle activity (median, static and peak electrical activity) is given for each of the assessed muscles including the relative difference between the two surgical techniques. Those relative differences were statistically significant for the left erector spinae muscle for all of the three characteristics of muscle activity with lower erector spinae muscle activation in RALS for median (61%), static (65%), and peak (49%) electrical activity, respectively. Statistically significant lower activities were also found in both splenius muscles (19–31% reduction) and in the right extensor digitorum muscle when RALS was applied (37–53% reduction). In contrast, the activity of the left trapezius muscle (non-dominant arm) was higher during RALS which revealed statistically significant results in the median (33% higher than in CLS) and peak electrical activity (48% higher than in CLS).

**Table 2:** Muscle activity profiles related to the applied operation method

Muscle		Median electrical activity (50th percentile)						Static electrical activity (10th percentile)						Peak electrical activity (90th percentile)					
		Mean (SD)		rel. diff [%]	Effect size d	F value	p value	Mean (SD)		rel. diff [%]	Effect size d	F value	p value	Mean (SD)		rel. diff [%]	Effect size d	F value	p value
		CLS	RALS					CLS	RALS					CLS	RALS				
Splenius capitis muscle activity [%RVE]	Left	28.65 (7.38)	23.09 (4.07)	**− 19,42**	**0.9**	**4.47**	**0.0471**	17.66 (4.95)	13.98 (3.14)	**− **20,84	0.9	4.14	0.0546	56.15 (14.36)	38.91 (9.25)	**− 30,70**	**1.4**	**11.22**	**0.0032**
	Right	29.52 (5.64)	24.97 (4.91)	**− **15,42	0.9	3.76	0.0663	20.17 (4.86)	14.59 (4.94)	**− 27,66**	**1.1**	**7.08**	**0.0150**	48.65 (12.76)	39.62 (8.25)	**−18,56**	**0.8**	**5.14**	**0.0346**
Trapezius descendens muscle activity [%RVE]	Left	14.36 (8.03)	19.10 (6.49)	**32,97**	**− 0.6**	**5.05**	**0.0361**	7.68 (5.64)	7.99 ± 3.35	4,04	**− **0.1	0.14	0.7169	22.65 (8.77)	33.54 (10.58)	**48,08**	**− 1.1**	**9.48**	**0.0059**
	Right	26.11 (11.07)	21.33 (9.32)	**− **18,33	0.5	1.54	0.2297	14.19 (7.67)	9.66 (5.56)	**− **31,92	0.7	4.14	0.0553	41.57 (12.91)	38.73 (13.37)	**− **6,83	0.2	0.25	0.6217
Extensor digitorum muscle activity [%RVE]	Right	64.98 (34.12)	40.73 (11.83)	**− 37,31**	**0.9**	**5.14**	**0.0346**	21.47 (14.63)	13.11 (6.88)	**− **38,94	0.7	2.98	0.0997	165.08 (85.68)	77.26 (26.60)	**− 53,20**	**1.4**	**11.19**	**0.0032**
Flexor carpi radialis muscle activity [%RVE]	Right	79.08 (78.46)	91.87 (81.42)	16,18	**− **0.2	0.12	0.7311	16.37 (20.29)	21.51 (21.90)	31,40	**− **0.2	0.35	0.5619	174.70 (141.33)	213.13 (123.64)	22,00	**− **0.3	0.46	0.5036
Erector spinae muscle activity [%RVE]	Left	65.83 (32.03)	25.41 (10.81)	**− 61,40**	**1.7**	**16.08**	**0.0007**	23.72 (17.09)	8.42 (3.54)	**−64,50**	**1.2**	**8.32**	**0.0091**	111.63 (41.97)	57.03 (19.74)	**− 48,91**	**1.6**	**15.34**	**0.0009**

*RALS* Robotic-assisted laparoscopic surgery, *CLS* Conventional laparoscopic surgery, *SD* Standard deviation, *RVE* Reference voluntary electrical activation, *rel. diff*. Relative difference (CLS-RALS)/CLS*100, bold letters indicate statistically significance (*p* < 0.05)

#### Secondary outcomes

##### Head, arm, and torso posture

Both surgical techniques required the surgeons to work with a slightly extended head posture with a median of 10° for CLS and 7° for RALS. Furthermore, peak head extension and flexion ranged from 17° to 4° in CLS and from 13° to 0° in RALS, with statistically significantly higher peak head extension in CLS (mean difference = 4°, *p* = 0.0259). The results of lateral head flexion indicated more left oriented head flexion during RALS with statistically significant findings for all three lateral head flexion parameters (Table 3). In addition, surgeons had to flex their torso more during CLS than during RALS, again with statistically significant differences for three trunk flexion parameters (Table 3). Arm posture also differed between the two surgical techniques with generally more arm anteversion for RALS and a higher arm abduction angle (median and peak) of the left arm in RALS compared to CLS. For the right arm peak arm abduction was higher in RALS (Table 3).

**Table 3:** Posture related to the applied operation method

Joint angle	50th percentile					10th percentile					90th percentile				
	Mean (SD) [°]		|diff| [°]	F value	p value	Mean (SD) [°]		|diff| [°]	F value	p value	Mean (SD) [°]		|diff| [°]	F value	p value
	CLS	RALS				CLS	RALS				CLS	RALS			
Head flexion/extension [negative values = extension]	− 9.99 (4.63)	− 6.92 (6.67)	3.07	2.66	0.1185	− 17.37 (5.21)	−13.25 (6.01)	**4.13**	**5.79**	**0.0259**	4.25 (8.38)	0.26 (6.29)	3.99	1.63	0.2165
Head lateral flexion [negative values = flexion to the right]	− 0.58 (3.51)	4.53 (6.27)	**5.11**	**12.58**	**0.0020**	− 6.87 (3.96)	− 2.18 (7.53)	**4.69**	**11.47**	**0.0029**	5.90 (3.48)	12.20 (5.08)	**6.30**	**13.36**	**0.0016**
Torso flexion/extension [positive values = flexion]	8.21 (2.18)	3.26 (3.27)	**4.95**	**21.15**	**0.0002**	3.60 (1.90)	0.60 (3.76)	**3**	**6.57**	**0.0185**	13.91 (3.31)	6.23 (3.48)	**7.68**	**31.32**	** < 0.0001**
Torso lateral flexion [negative values = flexion to the right]	− 3.87 (4.85)	− 5.94 (4.29)	2.07	3.43	0.0789	− 11.39 (4.78)	− 10.98 (4.23)	0.41	0.02	0.8920	4.96 (6.29)	− 1.31 (4.86)	**6.27**	**30.11**	** < 0.0001**
Arm adduction/abduction [positive values = abduction]															
Left	14.94 (6.23)	24.29 (10.42)	**9.35**	**13.56**	**0.0015**	0.33 (8.24)	14.14 (9.84)	**13.81**	**35.66**	** < 0.0001**	28.24 (6.42)	34.21 (10.47)	5.97	3.74	0.0675
Right	20.15 (12.11)	23.20 (8.19)	3.05	1.46	0.2416	6.80 (10.31)	15.36 (7.88)	**8.56**	**12.68**	**0.0020**	36.12 (11.13)	34.33 (10.69)	1.79	0.15	0.7007
Arm anteversion/retroversion [positive values = anteversion]															
Left	− 10.58 (5.86)	26.01 (3.82)	**36.59**	**345.62**	** < 0.0001**	− 24.92 (5.91)	16.55 (5.02)	**41.47**	**314.93**	** < 0.0001**	5.15 (9.26)	36.61 (3.54)	**31.46**	**142.98**	** < 0.0001**
Right	13.96 (23.82)	25.01 (5.05)	11.05	3.54	0.0747	− 8.11 (16.77)	14.97 (6.33)	**23.08**	**27.78**	** < 0.0001**	36.68 (21.76)	36.29 (6.02)	0.39	0.001	0.9732

*RALS* Robotic-assisted laparoscopic surgery, *CLS* Conventional laparoscopic surgery, *SD* Standard deviation, *|diff|* Absolute values of the difference (RALS–CLS), bold letters indicate statistically significance (*p* < 0.05)

##### Heart rate

The median heart rate was slightly higher for CLS (79 bpm) compared to RALS (72 bpm). The statistical analysis revealed this difference to be statistically significant (F: 4.05, *p* = 0.0383).

##### Perceived physical discomfort

During almost all 22 surgical procedures, no discomfort was reported by the surgeons. In only one surgery, there was a perceived discomfort rating in the neck area with an intensity level of 2 (0 = no discomfort, 10 = maximum discomfort).

##### Perceived workload

The results of this exploratory study indicated no differences in perceived mental or physical workload between the two surgical techniques (Table 4).

**Table 4:** Supplemental data to describe the procedures according to the applied surgical technique

Variable		Surgical technique	*25th percentile*	Median	*75th percentile*
Duration of surgical procedures	[min]	CLS	*22*	32	*50*
		RALS	*16*	32	*37*
Working heart rate	[1/min]	CLS	*67.9*	79.1	*100.3*
		RALS	*63.4*	72.3	*89.1*
Difficulty of surgical procedures	[0 – 100 mm]	CLS	*7*	34	*75*
		RALS	*9*	38	*63*
Working precision	[0 – 100 mm]	CLS	*98*	99	*100*
		RALS	*12*	55	*87*
Perceived workload	Mental demand [0 – 20]	CLS	*6*	12	*14*
		RALS	*10*	12	*16*
	Physical demand [0 – 20]	CLS	*4*	6	*12*
		RALS	*4*	6	*8*

*RALS* Robotic-assisted laparoscopic surgery, *CLS* Conventional laparoscopic surgery

##### Eyestrain

No eyestrain was reported in any of the observed surgeries.

#### Characterization of the performed surgical work

An overview of the supplemental data characterizing the included surgical procedures is presented in Table 4. The observed duration of the surgical procedures (excluding preparation time) was similar between CLS and RALS. However, please note that this duration does not reflect the total time for the surgical procedure or technique (including preparation time): operations with Senhance® generally took longer than CLS because it took considerably more time to correctly dock the robotic arms and insert the instruments prior to the start of the procedure. For the evaluation of ergonomics in this exploratory study, it was important that observation times were similar. Perceived difficulty of the surgical procedures was also similar between CLS (median = 34 mm) and RALS (median = 38 mm) whereby 0 mm indicated the lowest and 100 mm the highest difficulty. Working precision was rated higher for CLS with a median of 99 compared to RALS with a median of 58 mm (0 mm = lowest perceived working precision, 100 mm highest perceived working precision).

## Discussion

The primary aim of this exploratory study was to investigate the potential impact of the Senhance® Surgical System on surgeons’ muscular demands. The majority of the examined muscles showed a reduction in surgeons' muscular strain when using the surgical robot. In two muscles, there was no difference between the two surgical techniques, and in one muscle, the strain was lower under conventional procedures.

Most of the differences in muscular demands can be explained by the body posture required during the corresponding surgical technique. During RALS, head flexion and extension postures tended towards a more neutral position compared to CLS which reached statistical significance for the 10th percentile (peak head extension) explaining the lower activity of the bilateral splenius muscle. The lower erector spinae muscle activity during RALS can be explained by two posture observations. First, the median and peak trunk flexion (50th, 10th and 90th percentile) was 5° to 8° greater in CLS, respectively. Although these differences in trunk flexion between RALS and CLS can be considered as small and the trunk posture was generally rather upright for both surgical techniques, studies on the effects of trunk supporting exoskeletons suggest that muscle activity increases even at flexion angles that deviate only slightly from the upright posture [27, 28]. Second, Senhance® procedures are performed while the surgeon is seated in a chair with a backrest, whereas CLS requires the surgeon to stand. Although a comparison between standing and sitting without a backrest with identical spinal posture resulted in significantly greater activation of the erector spinae muscle when sitting [29], it has been shown that using a backrest when sitting significantly reduces activation of the erector spinae muscle compared to sitting without a backrest [30]. The use (duration and frequency) of the backrest while working with Senhance® was not monitored in the present study. However, it is likely that leaning on the back contributed to the reduced muscle activity in the lower back during RALS which suggests the advantage of a console/chair design with back support.

The reduced muscle activity in the right forearm extensor muscle cannot be explained by the postural data. Since the surgeons had to start the operation in a sterile field (insufflation of the pneumoperitoneum, incision and placement of trocars, docking of the instruments), we were not able to use posture sensors on the wrists or hands. However, laboratory studies on wrist postures during conventional laparoscopic surgery showed a significant amount of non-ergonomic postures in the right wrist both related to the surgeon–patient positioning and to the design of laparoscopic instruments [31, 32]. The instruments that are used with Senhance® are real laparoscopic instruments and hand pieces that are modified with specific adapters for docking, and may, therefore, not provide a benefit in terms of muscular demands in the lower arm itself. However, the surgeon’s position in the console may allow for a better adjustment of the hand-arm to handle position which may have resulted in a reduced muscle activity in the right extensor digitorum muscle during RALS.

Although working with Senhance® mainly relieved the muscles, there was an increase in muscle activity of the left trapezius muscle compared to CLS. Under CLS conditions, this left shoulder–neck muscle is only slightly activated due to the setting of the surgical procedures with a dominance of the right hand–arm system [15]. During RALS, both arms of the body are intended to be placed on the armrests of the console / chair (see Fig. 1), which leads to more arm anteversion and arm abduction, i.e., a different basic posture of the arms and shoulders, which can be attributed to the increase in left trapezius muscle activity. Mental demands also have the potential to increase the activity of the upper trapezius muscle [33], but perceived mental workload and task difficulty were rated similar between RALS and CLS. In contrast, Menke, et al. [34] found increased mental demands when using the Senhance® compared to CLS and suggested that a lack of routine in RALS may have contributed to this finding. The two included surgeons of the present study were very experienced and already used to work with other robotic systems, so it is possible that there was sufficient routine for the Senhance® procedures studied.

As a supplemental outcome to the objectively measured muscle strain, the level of perceived musculoskeletal discomfort has been assessed throughout each surgical procedure. In this respect, the subjects of the present study did not report any musculoskeletal discomfort during the observed procedures although discomfort levels may already increase in the course of a single laparoscopic procedure as shown by others [35]. It is likely that the short observation period with a median of 32 min in RALS and CLS may not be long enough to induce relevant musculoskeletal complaints, or that the two surgeons included in the present study represent some kind of positive selection in terms of being experienced and resilient to their working conditions.

A secondary aim of this study was to compare the perceived eye strain and muscle activity of the bilateral splenius capitis muscle between the two surgical techniques CLS and RALS. This aim was motivated by a novel feature of the Senhance® System including multiple modalities for camera control. These include instrument-guided camera positioning (“Follow Us” and “Go To”), maintaining targeted field of view while zooming (“Smart Zoom”) and “Eye Tracking” which allows the surgeon to control the camera with their eyes. The eye tracker allows the surgeon to zoom in and out of the operating field on the screen and select functions of the console menu by eye movements; however, there are recent studies on such technological innovations and head-mounted displays demonstrating negative effects such as muscle and eye fatigue in users [36]. In our study, bilateral splenius activity was not impaired during RALS, and no eyestrain was reported in any of the surgical procedures. However, given the short observation times (median of 32 min) of the present study which may be too short for inducing relevant levels of neck or eyestrain, this aspect should be addressed by future studies with longer observation period.

Finally, as part of our experimental approach, we also assessed heart rate as an indicator of cardiopulmonary stress. The heart rate was lower in the RALS group which can be explained by a decreased burden for the aforementioned muscles, however these data indicated that cardiopulmonary stress played a subordinate role, as it was well below the continuous performance limit for physical work lasting up to an 8-h shift [37] for both types of surgery in an overall limited number of cases with minor complexity.

### Limitations

This exploratory study has several limitations that have to be addressed. One is the fact that all 23 surgical procedures were performed by only 2 male surgeons and the duration of the observed surgical procedures was about 30 min and therefore rather short. The results of the present study cannot be generalized and should be understood as initial indications of how the muscular demand situation could change when working with Senhance®. Follow-up studies including larger samples are required. In addition, it can be discussed if a confident plateau in the learning curve of the two surgeons with Senhance® was achieved before the start of this study, since perceived working precision was lower during RALS. A systematic review comparing CLS and RALS suggests that muscular demands in the trapezius muscle may depend on surgeon’s level of experience with the robot [38]. However, this would rather result in an underestimation of the relief in muscle demands by Senhance® since incomplete motor learning is likely to result in increased levels of muscle activity [39].

For safety reasons, we decided not to normalize the electrical activity to a reference activity obtained during voluntary isometric maximum muscle contraction, since we investigated the surgeons prior and during the operations of their patients. A maximum voluntary contraction of the muscle—even if this is a standard in laboratory studies—always represents a certain risk of injury to the affected muscle or joint structure. Therefore, our results were normalized to submaximal reference contraction which allow us to interpret differences in muscular demands between the surgical techniques but do not allow to describe the level of muscle activation in terms of the muscle’s maximum capacity.

## Conclusion

This exploratory study identified a potential of the Senhance® Surgical System for ergonomic improvements which may lower the muscular demands on the surgeon and indicated no impairments by the novel eye-tracking feature on neck muscle demands and eyestrain in short-term application. Follow-up studies with larger and more diverse study individuals could use our initial results of this present study as a basic reference, as they provide a suggestion of parameters in terms of the body regions that are likely to benefit from the Senhance® System and which may be impaired. Furthermore, the clinical outcomes and aspects relating to the integration of the robot into everyday clinical practice should be taken into account to gain a more comprehensive picture of the potential of this robotic system.

## Supplementary Information

Below is the link to the electronic supplementary material.

Supplementary file1 (DOCX 15 KB)

Supplementary file2 (DOCX 16 KB)

Supplementary file3 (DOCX 16 KB)

## Data Availability

Data are provided within the manuscript or supplementary information files.
