# Supplementary material for: Ergonomic evaluation of the Senhance® robotic system in minimally invasive gynecologic procedures versus conventional laparoscopy: an exploratory study focusing on surgeon’s muscle activity
Source: Arch Gynecol Obstet. 2026 Jan 16;313(1):45. doi: 10.1007/s00404-025-08292-0 (PMC12811308; doi:10.1007/s00404-025-08292-0)
Supplement: Supplementary file 1 — Supplementary file1 (DOCX 15 KB) [file 404_2025_8292_MOESM1_ESM.docx]

**Supplemental material A - Isometric muscle contractions with submaximal force application for determining the reference muscle activity**

1. Left and right descending trapezius muscles: The subject stood upright with a shoulder-width stance and held a 2 kg dumbbell in each hand for 20 seconds. The arms were in 90° abduction in the scapular plane, the elbows were extended and the palms were facing downwards.
2. Extensor digitorum muscle: The subject sat upright on a chair and held the forearm in the air. The upper arm hung vertically down along the body, the elbow was flexed at 90° and the forearm pointed straight forward. The palm of the hand, stretched forward parallel to the forearm, pointed downwards. There was a loop on the back of the subject's hand at the level of the proximal interphalangeal joint, which was connected to a 2 kg dumbbell under the hand. The subject held this with an outstretched hand for 20 seconds.
3. Flexor carpi radialis muscle: The subject sat upright on a chair and held the forearm in the air. The upper arm hung vertically down along the body, the elbow was flexed at 90° and the forearm pointed straight forward. The palm of the hand, stretched forward parallel to the forearm, pointed upwards. There was a loop on the back of the subject's hand at the level of the proximal interphalangeal joint, which was connected to a 2 kg dumbbell under the hand. The subject held this with an outstretched hand for 20 seconds.
4. Erector spinae and splenius capitis muscle: The subject sat upright on a chair without a backrest. The knees were at a 90-degree angle. The arms hung down loosely along the body without holding on. A device with a pulley was attached to a table. The subject was seated in front of this table facing the device. A sling was attached to the subject's head, which was connected to a cable that was passed over the pulley of the device. The subject was instructed to keep the head and the entire upper body upright. An eight kg weight was carefully attached to the end of the cable to create a pull, and the subject had to counteract flexion in the trunk and neck by contracting the erctor spinae and splenius capitis muscles for a period of about 20 seconds.
