# Supplementary material for: Ergonomic evaluation of the Senhance® robotic system in minimally invasive gynecologic procedures versus conventional laparoscopy: an exploratory study focusing on surgeon’s muscle activity
Source: Arch Gynecol Obstet. 2026 Jan 16;313(1):45. doi: 10.1007/s00404-025-08292-0 (PMC12811308; doi:10.1007/s00404-025-08292-0)
Supplement: Supplementary file 2 — Supplementary file2 (DOCX 16 KB) [file 404_2025_8292_MOESM2_ESM.docx]

**Supplemental material B – Order of performed surgical procedures**

| Subject 1 | | Subject 2 | |
| --- | --- | --- | --- |
| Surgical technique | Experimental days | Surgical technique | Experimental days |
| CLS 1 | Day 1 | CLS 1 | Day 1 |
| CLS 2 |  | CLS 2 |  |
| CLS 3 | Day 2 | CLS 3 | Day 2 |
| CLS 4 |  | CLS 4 |  |
| RALS 1 | Day 3 | CLS 5 | Day 3 |
| RALS 2 |  | CLS 6 |  |
| RALS 3 | Day 4 | RALS 1 | Day 4 |
| CLS 5 |  | RALS 2 |  |
| RALS 4 | Day 5 | RALS 3 | Day 5 |
| RALS 5 |  | RALS 4 | Day 6 |
|  |  | CLS 7  RALS 5 | Day 7 |
|  |  | RALS 6 | Day 8 |

CLS conventional laparoscopic surgery; RALS robotic assisted laparoscopic surgery
