# Supplementary material for: Ergonomic evaluation of the Senhance® robotic system in minimally invasive gynecologic procedures versus conventional laparoscopy: an exploratory study focusing on surgeon’s muscle activity
Source: Arch Gynecol Obstet. 2026 Jan 16;313(1):45. doi: 10.1007/s00404-025-08292-0 (PMC12811308; doi:10.1007/s00404-025-08292-0)
Supplement: Supplementary file 3 — Supplementary file3 (DOCX 16 KB) [file 404_2025_8292_MOESM3_ESM.docx]

**Supplemental material C – Type of performed surgical procedures**

| **Number of procedures*** | **Type of laparoscopic procedures** |
| --- | --- |
| 4 | Hysterectomie |
| 20 | Adnexal surgery (*uni- or bilateral*) |
|  | - Salpingectomy (**4x**) |
|  | - Ovarian cyst excision (**9x**) |
|  | - Salpingo-oophorectomy (**7x**) |
| 5 | Adhesiolysis (peritoneal, intestinal) |
| 3 | Endometriosis (uterosacral ligament, pelvic side wall) |

* *multiple procedures could be carried out during one surgery*
